# Diagnostic value of one-stop CT energy spectrum and perfusion for angiogenesis in colon and rectum cancer

**DOI:** 10.1186/s12880-024-01291-8

**Published:** 2024-05-21

**Authors:** Ling Zhao, Wei Zhou, Yu Fu, Yanlei Ge, Li Feng, Xingwen Wang, Zemao Li, Weibin Chen

**Affiliations:** North China University Of Science And Technology Affiliated Hospital, Tangshan, Hebei 063000 China

**Keywords:** Energy spectrum and perfusion CT parameters, Microvessel density (MVD), Colorectal cancer

## Abstract

**Objective:**

Evaluation of the predictive value of one-stop energy spectrum and perfusion CT parameters for microvessel density (MVD) in colorectal cancer cancer foci.

**Methods:**

Clinical and CT data of 82 patients with colorectal cancer confirmed by preoperative colonoscopy or surgical pathology in our hospital from September 2019 to November 2022 were collected and analyzed retrospectively. Energy spectrum CT images were measured using the Protocols general module of the GSI Viewer software of the GE AW 4.7 post-processing workstation to measure the CT values of the arterial and venous phase lesions and the neighboring normal intestinal wall in a single energy range of 40 kev∼140 kev, and the slopes of the energy spectrum curves (λ) were calculated between 40 kev-90 kev; Iodine concentration (IC), Water concentration (WC), Effective-Z (Eff-Z) and Normalized iodine concentration (NIC) were measured by placing a region of interest (ROI) on the iodine concentration map and water concentration map at the lesion and adjacent to the normal intestinal wall.Perfusion CT images were scanned continuously and dynamically using GSI Perfusion software and analyzed by applying CT Perfusion 4.0 software.Blood volume (BV), blood flow (BF), surface permeability (PS), time to peak (TTP), and mean transit time (MTT) were measured respectively in the lesion and adjacent normal colorectal wall. Based on the pathological findings, the tumors were divided into a low MVD group (MVD < 35/field of view, *n* = 52 cases) and a high MVD group (MVD ≥ 35/field of view, *n* = 30 cases) using a median of 35/field of view as the MVD grouping criterion. The collected data were statistically analyzed, the subjects’ operating characteristic curve (ROC) was plotted, and the area under curve (AUC), sensitivity, specificity, and Yoden index were calculated for the predicted efficacy of each parameter of the energy spectrum and perfusion CT and the combined parameters.

**Results:**

The CT values, IC, NIC, λ, Eff-Z of 40kev∼140kev single energy in the arterial and venous phase of colorectal cancer in the high MVD group were higher than those in the low MVD group, and the differences were all statistically significant (*p* < 0.05). The AUC of each single-energy CT value in the arterial phase from 40 kev to 120 kev for determining the high or low MVD of colorectal cancer was greater than 0.8, indicating that arterial stage has a good predictive value for high or low MVD in colorectal cancer; AUC for arterial IC, NIC and IC + NIC were all greater than 0.9, indicating that in arterial colorectal cancer, both single and combined parameters of spectral CT are highly effective in predicting the level of MVD. The AUC of 40 kev to 90 kev single-energy CT values in the intravenous phase was greater than 0.9, and its diagnostic efficacy was more representative; The AUC of IC and NIC in venous stage were greater than 0.8, which indicating that the IC and NIC energy spectrum parameters in venous stage colorectal cancer have a very good predictive value for the difference between high and low MVDs, with the greatest diagnostic efficacy in IC.The values of BV and BF in the high MVD group were higher than those in the low MVD group, and the differences were statistically significant (*P* < 0.05), and the AUC of BF, BV, and BV + BF were 0.991, 0.733, and 0.997, respectively, with the highest diagnostic efficacy for determining the level of MVD in colorectal cancer by BV + BF.

**Conclusion:**

One-stop CT energy spectrum and perfusion imaging technology can accurately reflect the MVD in living tumor tissues, which in turn reflects the tumor angiogenesis, and to a certain extent helps to determine the malignancy, invasion and metastasis of living colorectal cancer tumor tissues based on CT energy spectrum and perfusion parameters.

## Background

Disease spectrum and dietary structure have gradually changed in China, making the incidence of colorectal cancer on the rise year by year [1], and it is one of the key elements of cancer prevention and treatment in China. Colorectal cancer is mainly treated with surgery in the early stage, but many patients are already in the advanced stage of the disease when they are diagnosed, and metastasis to distant tissues or organs may also occur. Studies have confirmed that the earlier colorectal cancer is diagnosed and treated, the better the prognosis for patients [2]. Abnormal growth of malignant tumor cells requires special nutrition and oxygen supply, so the growth of blood vessels in tumor tissues is indispensable, but compared with the blood vessels of normal tissues, the newborn blood vessels are deficient in their structural integrity, and the probability of tumor cells penetrating the blood vessels is higher. Therefore, the primary prerequisite for tumor invasion and metastasis is the presence of tumor angiogenesis. Based on this, the aim of this study was to explore the predictive value of one-stop CT energy spectroscopy and perfusion combined examination for angiogenesis in colorectal cancer.

## Objects and methods

### Objects

Clinical and CT data were collected from patients with colorectal cancer who presented to our hospital from September 2019 to November 2022, all of whom had preoperative pathological confirmation of colorectal cancer by colonoscopic forceps examination, followed by one-stop scanning with CT energy spectroscopy and perfusion imaging, and none of whom had undergone any treatment prior to the examination, with the surgery needing to be completed within 14 days of the CT examination. A total of 82 patients were collected, including 48 males and 34 females, with an age range of 45–79 years and an average age of (67.94 ± 6.56) years, and all the patients were analyzed retrospectively. This study was reviewed and approved by the Hospital Ethics Committee.

#### Inclusion Criteria

1.  The patient’s clinical data, pathology and imaging are complete.
2.  The patient’s pre-operative colonoscopy forceps for pathology was colorectal cancer, and the post-operative pathology was also colorectal cancer.
3.  The patient did not undergo any relevant treatment prior to surgery.
4.  The patient had no history of iodine contrast allergy, and the CT examination was successfully completed.
5.  Patients signed informed consent.

#### Exclusion Criteria

1.  The patient had severe liver and renal impairment and severe cardiovascular and cerebrovascular disease [3–5].
2.  Patients who had undergone clinical therapeutic intervention for colorectal cancer before the examination or experienced tumor recurrence.
3.  Patients with more than 1 month between CT energy spectrum and perfusion examination and surgical resection.
4.  Those who are unable to obtain accurate energy spectra and perfusion parameters due to artifacts, etc.

Cluster situation: The median microvessel count of 35/field of view was used as the grouping criterion, and MVD < 35/field of view was regarded as the low MVD group, with a total of 52 cases; MVD ≥ 35 was regarded as the high MVD group, with a total of 30 cases.

### Patient Preparation for examination

1.  All patients were required to exclude patients’ history of allergies, contraindications, and prohibited medications before performing CT energy spectrum and perfusion scans.
2.  Patients are advised not to do gastrointestinal imaging within one week before the examination to avoid artifacts; Eat soft food 2 days before the test, and bowel cleansing and water fasting for 6∼8 h the night before the examination.
3.  Inform the patient of the entire examination process and precautions before the examination to eliminate the patient’s nervousness and to get good cooperation.
4.  The patient will be instructed to remove metal objects from the body, and the un-scanned area will be covered with a lead jacket, and the patient will be radiologically protected.
5.  Scanning position is supine with hands raised above the head.

### Inspection methods

Inspection of equipment and medicines: Adopting the GE Revolution CT produced by the U.S. GE Company and the image post-processing workstation AW 4.7, which is compatible with it. The contrast agent was iohexol 300 mgI/mL with 0.9% saline. The contrast syringe was ORICH double barrel high pressure syringe from Germany.

Inspection methods: Conventional CT scanning was performed first to determine the site and extent of the lesion, and then one-stop energy spectrum and perfusion CT dual-phase enhancement scanning was performed. The scanning parameters were as follows: tube voltage 100kvp, tube current 80 mA, rotation time 0.5s during perfusion; spectrum scanning tube voltage high and low energy (80∼140kvp) instantaneous switching, automatic tube current, rotation time 0.5s; pitch 0.992, matrix 512 × 512, layer thickness 1.25 mm, spacing 1.25 mm. Iohexol (300 mgI/mL) was injected at a dose of 1.1 mL/kg, at a rate of 4.0 mL/s, and 25 mL of additional saline was added to each of the contrasting doses after completion of injection. The one-stop CT energy spectrum and perfusion imaging scan is divided into 4 scanning protocols: 1 and 3 scanning protocols were perfusion, corresponding to the perfusion inflow and outflow periods, respectively, and a total of 18 images were acquired in the inflow and outflow periods; 2 and 4 scanning protocols were the arterial and venous phases of the energy spectrum. Scanning began 5s after contrast injection, every 2.5s, at the end of the 8th time-phase, arterial-phase spectral images were scanned, and then the 10th time-phase to the 17th time-phase were scanned, followed by venous-phase spectral images, and then CT perfusion images and arterial and venous-phase spectral images were obtained. The original energy spectrum image was reconstructed with a layer thickness of 1.25 mm and a layer spacing of 1.25 mm, and after reconstruction was completed, it was transferred to the GE AW 4.7 post-processing workstation.

Image post-processing and data acquisition: All images were transferred to GE AW 4.7 post-processing workstation, and GSI Viewer analysis software was applied to observe and analyze the spectral images and measure various spectral parameters. Selected Spectral HU Curve. Criteria for selecting ROI: ROI placement of the lesion area, the largest cross-section of the lesion was selected for measurement by MPR reconstruction, and the solid portion of the tumor lesion area was selected and manually outline the ROI area by enhancing the image to avoid outlining blood vessels, necrotic liquefaction areas, gas, and fat; and copying and pasting were selected to ensure that the ROIs sketched were of the same size and the same location. By plotting ROIs in the tumor lesion area, 40 keV∼140 keV single-energy CT values were recorded. The IC and WC as well as Eff-Z of different stages of tumor lesion areas at the same level of iodine-based and water-based maps as well as effective atomic number sections were measured using ROI copy-paste.

Due to individual patient differences, the IC was standardized for different staged tumor lesion areas. The abdominal aorta or iliac artery at the same level of the measured tumor lesion area was used as a reference to measure the iodine-based value in the artery, and the ROI was selected to be about 1/2 of the internal diameter of the artery. Equations 1 and 2 were used to calculate the NIC and λ of the tumor lesion areas at different stages.

1 $$\eqalign{& {\text{NIC = IC lesion area OR }} \cr & {\text{normal intestinal wall/IC homozygous aorta}} \cr}$$

2 $$\lambda {\text{ = }}\left( {{\text{CT}}\,{\text{value}}\,{\text{40kev - CT}}\,{\text{value}}\,{\text{90kev}}} \right){\text{/50kev}}$$

Perfusion images were analyzed using CT Perfusion software, which used a tumor perfusion model, selected inflow target arteries and outflow target veins, and automatically fited the TDC to derive perfusion pseudo-color maps. ROIs were labeled at 2 to 3 levels of the cancer lesion, respectively, to obtain the corresponding perfusion parameters. That is BV, BF, PS, TTP, MTT in the tumor lesion area.

The above parameters (40kev∼140kev single energy CT value, WC, IC, NIC,λ, Eff-Z, BV, BF, PS, TTP, MTT) were averaged over 3 measurements in the same tumor lesion area. The ROI selection process was guided by two senior imaging physicians, and agreement was reached through negotiation in case of disagreement.

### Pathological examination

The pathology specimens obtained after surgery were routinely processed, placed in formalin for soaking, cut the lesion tissue for paraffin fixation and embedding, and then processed for paraffin block sectioning with a thickness of 4 μm, and the specimens were stained using conventional HE methods. Pathology review was conducted by two senior chief physicians in a double-blind manner, observing all stained sections under high and low magnification light microscopes, when disagreement was encountered between the two, agreement was reached after consultation to determine whether the lesion was colorectal cancer or not.

### Microvascular count

Immunohistochemical labeling was performed using the CD34 monoclonal. MVD counting principles [6]: (1) Individual endothelial cells or clusters of endothelial cells stained brown and not associated with adjacent microvessels, tumor cells and other tissues were counted as one microvessel; (2) Microvessels that may belong to the same vessel but are in different locations were treated as separate microvessels; (3) Vessels with a lumen area greater than 8 erythrocytes or a thick muscular layer were not counted as microvessels; (4) Microvessel counts were not performed in areas where cancer cells are sparse, such as adjacent normal tissue, tumor sclerosis or fibrosis.In each section, areas of high blood vessel density were selected in the low magnification (40×) field of view, and then microvessels were counted in the high magnification (100×) field of view. 3 high magnification fields of view were observed in each section, and the average value was taken as the final result of microvessel counting.

### Statistical methods

SPSS 21.0 and MedCalc statistical software were applied and measurement data were expressed as $\overline x \; \pm \;s$.The energy spectrum parameters and perfusion parameters between the two groups were subjected to normality test and variance chi-square analysis; if the data were normally distributed and the variance was chi-square, two independent samples t-test was performed; if the data were not normally distributed or the variance was not chi-square, nonparametric test was performed. Based on the results of the variability analysis, the energy spectrum parameters and perfusion parameters with statistically significant differences were modeled with binary logistic regression to obtain statistically significant parameters and joint parameter equations. The Receiver operation characteristic (ROC) curve was plotted, and the area under the curve (AUC) of the energy spectrum parameter, the perfusion parameter and the combined parameter were calculated (AUC criteria: 0.5∼0.7 diagnostic efficacy is average; 0.7∼0.8 diagnostic efficacy is moderate; greater than 0.8 diagnostic efficacy is better), so as to the diagnostic efficacy of energy spectrum parameters, perfusion parameters and combined parameters in diagnosing colorectal cancer, as well as the sensitivity, specificity, Youden’s index and the optimal threshold of the parameters were obtained. The test level of α = 0.05 was used.

## Results

General data of 82 patients with colorectal cancer were analyzed: 48 males, 34 females; age 45–79 years, mean (67.94 ± 6.56) years.

### Analysis of 40kev∼140kev single-energy CT values and energy spectrum parameters in the arterial phase of colorectal cancer with different microvessel densities and evaluation of diagnostic efficacy

By comparing the single-energy CT values of 40 kev∼140 kev in the arterial phase of colorectal cancer with different microvessel densities, it was found that all single-energy CT values of colorectal cancer in the high MVD group were higher than those in the low MVD group, the differences were statistically significant (both *P* < 0.05). See Table 1.

**Table 1:** Comparison of 40 keV∼140 keV single-energy CT values in arterial phase of colorectal cancer with different microvessel densities ($\overline x \; \pm \;s$,HU)

Single energy level/kev	Low MVD group/(*n* = 52)	High MVD group/(*n* = 30)	t	*P*
40	144.18 ± 18.84	197.90 ± 22.00	−11.69	0.001
50	106.36 ± 11.66	139.22 ± 15.35	−10.93	0.001
60	83.01 ± 8.00	106.63 ± 9.74	−11.88	0.001
70	68.55 ± 6.41	85.83 ± 7.50	−11.04	0.001
80	59.61 ± 6.15	72.72 ± 6.26	−9.24	0.001
90	53.60 ± 6.53	64.21 ± 6.10	−7.25	0.001
100	48.96 ± 6.25	58.17 ± 5.94	−6.55	0.001
110	46.56 ± 7.84	53.78 ± 6.13	−4.33	0.001
120	43.98 ± 6.65	50.85 ± 6.29	−4.59	0.001
130	42.25 ± 6.80	48.52 ± 6.43	−4.10	0.001
140	40.92 ± 6.89	46.39 ± 6.51	−3.53	0.001

Comparison of arterial-phase energy spectral parameters (WC, IC, NIC, λ, Eff-Z) in colorectal cancers with different microvascular densities showed that, except for WC, which was not statistically different (*P* > 0.05), the values of IC, NIC, λ, and Eff-Z were statistically different in colorectal cancers of the high and low MVD groups (all *P* < 0.05), and the values of each of the energy spectral parameters of colorectal cancers of the high MVD group were higher than those of the low MVD group. See Table 2.

One-way logistic regression analysis of IC, NIC, λ, and Eff-Z showed that IC, NIC, λ, and Eff-Z were all influencing factors of microvessel density in colorectal cancer (all *P* < 0.05); and the OR value of each parameter was greater than 1, which indicated that the probability of prediction of colorectal cancer was also relatively high for each 1-unit increase of each parameter respectively; however, it was found that the results of λ, Eff -Z’s confidence intervals were too large, which led to unstable results, so they were not investigated further. See Table 3.

**Table 2:** Comparison of arterial phase energy spectrum parameters in colorectal cancer with different microvessel densities ($\overline x \; \pm \;s$)

Spectral parameter	Low MVD group/(*n* = 52)	High MVD group/(*n* = 30)	t	*P*
WC	1031.79 ± 8.77	1032.41 ± 6.01	−0.34	0.732
IC	16.38 ± 3.44	24.07 ± 4.63	−8.57	0.001
NIC	0.18 ± 0.04	0.26 ± 0.04	−8.04	0.001
λ	2.23 ± 0.46	2.98 ± 0.46	−7.17	0.001
Eff-Z	8.56 ± 0.20	8.91 ± 0.29	−5.78	0.001

*Note* WC in µgI/mL; IC in µgI/mL

**Table 3:** One-way Logistic regression analysis of arterial phase energy spectrum parameters in colorectal cancer with different microvessel densities

Parametric	$\widehat{\varvec{\beta }}$	SE	Wald${\varvec{\chi }}^{2}$	*P*	OR	95% CI
IC	0.763	0.183	17.362	0.001	2.146	1.498–3.073
NIC	0.494	0.112	19.446	0.001	1.639	1.316–2.042
λ	3.664	0.823	19.581	0.001	38.229	7.612−191.992
Eff-Z	6.002	1.497	16.076	0.001	40.404	21.494−759.508

*Note* WC in µgI/mL; IC in µgI/mL

**Fig. 1:**
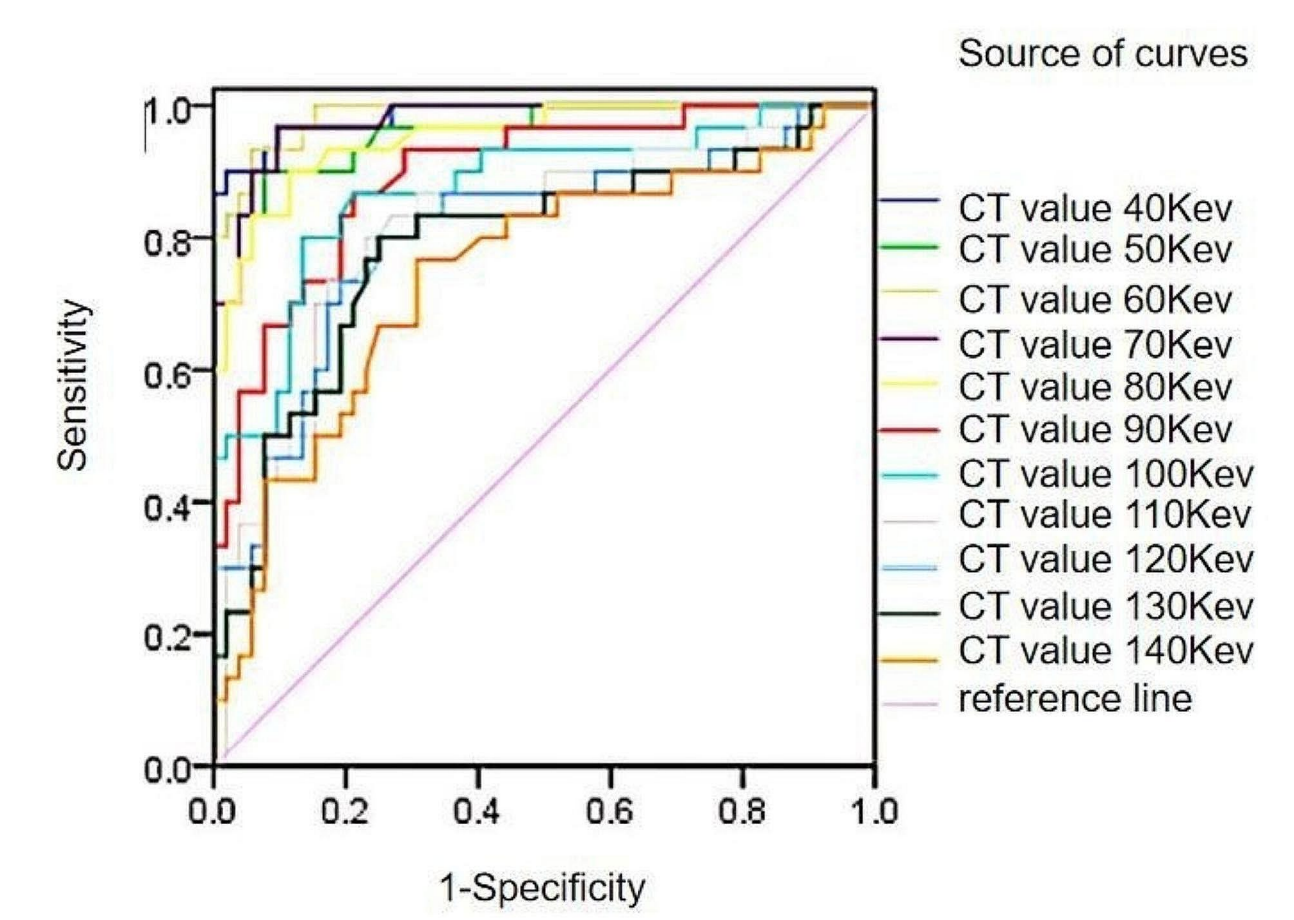
ROC curves of 40kev∼140kev single-energy CT values in arterial phase of colorectal cancer with different microvessel densities

The ROC curves of 40kev∼140kev single-energy CT values in arterial stage of colorectal cancer with different microvessel densities showed that the AUC of 40kev∼120kev single-energy CT values were all greater than 0.8, which indicated that the 40kev∼120kev single-energy CT images had better diagnostic efficacy for the differences in microvessel density in arterial stage of colorectal cancer, the efficacy of 40kev∼80kev single-energy CT values was more representative., among which 40kev and 60kev single-energy CT values have the largest area under the curve, with high sensitivity, specificity and Youden index. See Table 4; Fig. 1.

**Fig. 2:**
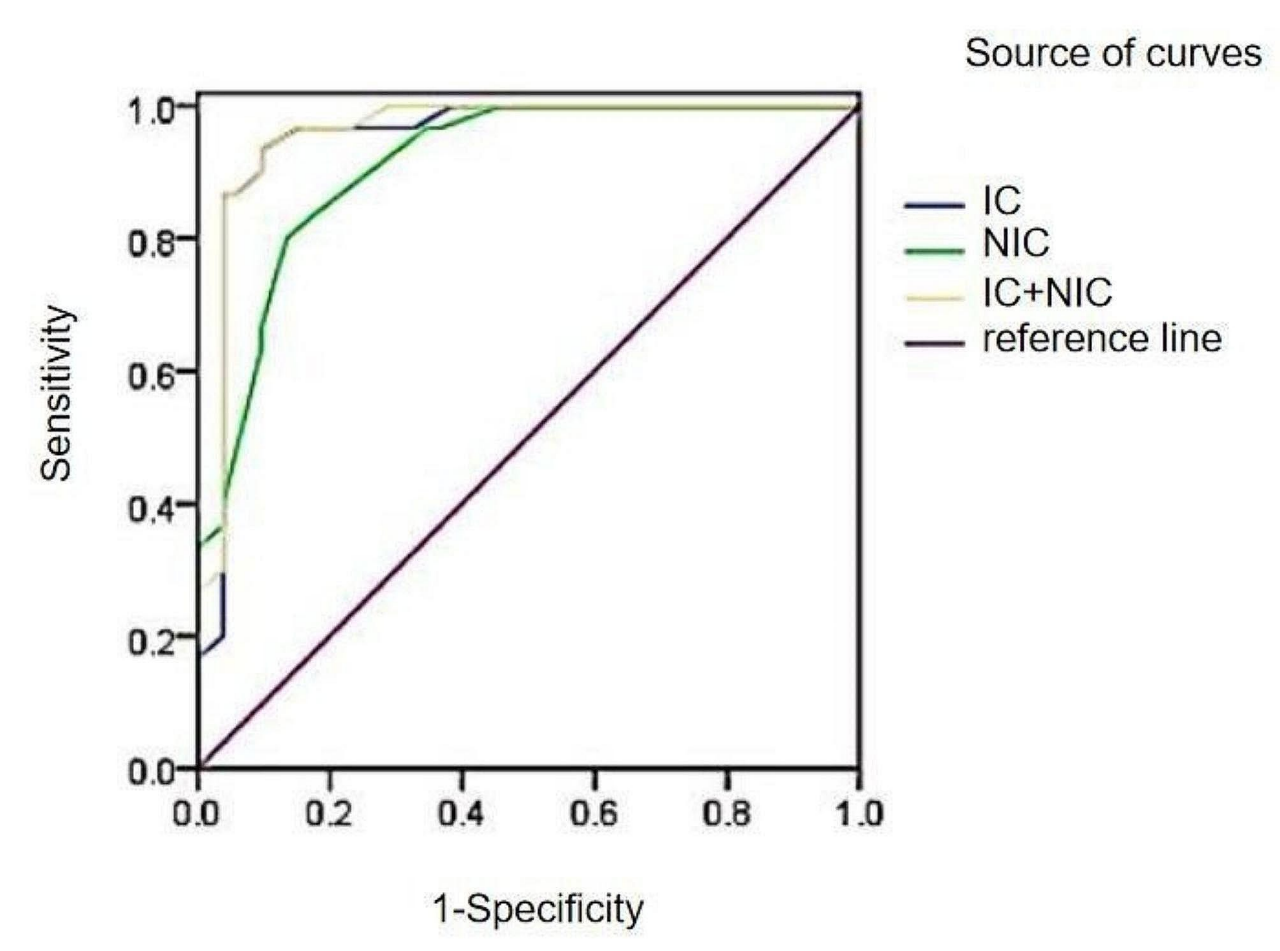
ROC curves of IC, NIC, and IC + NIC in arterial stage of colorectal cancer with different microvessel densities

The ROC curves of IC, NIC and IC + NIC in the arterial stage of colorectal cancer with different microvessel densities showed that the AUC of IC, NIC and IC + NIC.

were all greater than 0.9, which indicated that IC, NIC, IC + NIC spectroscopic parameters and the combination of them had excellent diagnostic efficacy in the arterial stage of colorectal cancer in the display of the differences in the microvessel densities, the best diagnostic efficacy was obtained with IC + NIC, but its sensitivity, specificity and Youden index were the same as that of IC., indicating that the judgment of the microvessel density of colorectal cancer was slightly improved in the case of the combined application of IC and NIC. See Table 5; Fig. 2.

**Table 4:** Area under the curve analysis of 40kev∼140kev single energy CT values in arterial phase of colorectal cancer with different microvessel densities

Single energy level/kev	AUC(95% CI)	Threshold/HU	Sensitivity/%	Specificity/%	Youden index
40	0.985(0.929–0.999)	174.91	90.00	98.08	0.8808
50	0.963(0.896–0.992)	121.56	90.00	92.31	0.8231
60	0.985(0.929–0.999)	95.12	93.33	94.23	0.8756
70	0.976(0.915–0.997)	74.81	96.67	90.38	0.8705
80	0.952(0.881–0.987)	65.56	90.00	88.46	0.7846
90	0.893(0.805–0.950)	57.91	86.67	78.85	0.6551
100	0.873(0.782–0.937)	55.09	80.00	86.54	0.6654
110	0.815(0.714–0.892)	50.38	80.00	76.92	0.5692
120	0.805(0.702–0.884)	47.81	80.00	75.00	0.5500
130	0.787(0.682–0.870)	46.18	80.00	75.00	0.5500
140	0.745(0.637–0.835)	43.50	76.67	69.23	0.4590

**Table 5:** Analysis of arterial-phase energy spectral parameters and area under the curve of combined parameters in colorectal cancer with different microvessel densities

Parametric	AUC(95% CI)	Threshold/HU	Sensitivity/%	Specificity/%	Youden index
IC	0.952(0.881–0.987)	19.32	93.33	90.38	0.8372
NIC	0.910(0.827–0.962)	0.22	80.00	86.54	0.6654
IC + NIC	0.959(0.891–0.990)	—	93.33	90.38	83.72

### Analysis of 40kev∼140kev single-energy CT values and energy spectral parameters and evaluation of diagnostic efficacy of colorectal cancer with different microvessel densities at venous stage

By comparing the single-energy CT values of 40 kev∼140 kev in the venous stage of colorectal cancer with different microvessel densities, it was found that all single-energy CT values of colorectal cancer in the high MVD group were higher than those in the low MVD group, the differences were statistically significant (all *P* < 0.05). See Table 6.

By comparing the venous phase energy spectral parameters (WC, IC, NIC, λ, Eff-Z) of colorectal cancer with different microvessel densities, it was shown that, except for WC, which was not statistically different (*P* > 0.05), the values of IC, NIC, λ, and Eff-Z were statistically different in colorectal cancer of the high and low MVD groups (both *P* < 0.05), and the values of each of the energy spectral parameters of colorectal cancer in the high MVD group were higher than those of the low MVD group. See Table 7.

One-way logistic regression analysis of IC, NIC, λ, and Eff-Z showed that IC, NIC, λ, and Eff-Z were all influencing factors of microvessel density in colorectal cancer (all *P* < 0.05); and the OR value of each parameter was greater than 1, indicating that for every 1-unit increase in each parameter respectively, the probability of predicting a high or low microvessel density in colorectal cancer was also relatively high; however, by observing the results It was found that the 95% confidence interval of Eff-Z was too large, which led to unstable results, so it was not studied further. See Table 8.

**Table 6:** Comparison of 40kev∼140kev single-energy CT values for different microvessel densities in venous stage colorectal cancer ($\overline x \; \pm \;s$,HU)

Single energy level/kev	Low MVD group/(*n* = 52)	Hign MVD group/(*n* = 30)	t	*P*
40	168.88 ± 26.97	238.59 ± 23.29	−11.83	0.001
50	123.72 ± 16.22	166.62 ± 13.33	−12.28	0.001
60	95.44 ± 10.41	128.27 ± 13.92	−12.13	0.001
70	77.63 ± 7.29	100.02 ± 10.81	−11.19	0.001
80	66.56 ± 6.04	82.36 ± 8.51	−9.79	0.001
90	59.12 ± 5.69	70.67 ± 6.69	−8.30	0.001
100	53.92 ± 5.70	62.83 ± 6.19	−6.60	0.001
110	50.28 ± 5.89	57.09 ± 5.97	−5.02	0.001
120	47.82 ± 6.06	53.02 ± 5.75	−3.81	0.001
130	45.76 ± 6.25	50.19 ± 5.47	−3.23	0.001
140	44.20 ± 6.41	48.82 ± 8.09	−2.85	0.001

**Table 7:** Comparison of energy spectrum parameters of colorectal cancer in venous stages with different microvessel densities ($\overline x \; \pm \;s$)

Parametric	Low MVD group/(*n* = 52)	High MVD group/(*n* = 30)	t	*P*
WC	1032.64 ± 7.09	1032.58 ± 4.59	−0.53	0.958
IC	20.27 ± 2.83	25.04 ± 1.81	−8.27	0.001
NIC	0.49 ± 0.09	0.63 ± 0.09	−5.89	0.001
λ	2.76 ± 0.49	3.24 ± 0.48	−4.34	0.001
Eff-Z	8.79 ± 0.23	9.03 ± 0.14	−5.24	0.001

**Table 8:** One-way logistic regression analysis of venous phase energy spectrum parameters in colorectal cancer with different microvessel densities

Parameter	$\widehat{\varvec{\beta }}$	SE	Wald${\varvec{\chi }}^{2}$	*P*	OR	95% CI
IC	0.936	0.222	17.848	0.001	2.549	1.652–3.936
NIC	0.134	0.031	19.045	0.001	1.144	1.077–1.215
λ	2.173	0.604	12.943	0.001	8.787	2.689–28.711
Eff-Z	6.615	1.732	14.593	0.001	74.606	25.053−2221.465

*Note* WC in µgI/mL; IC in µgI/mL

**Fig. 3:**
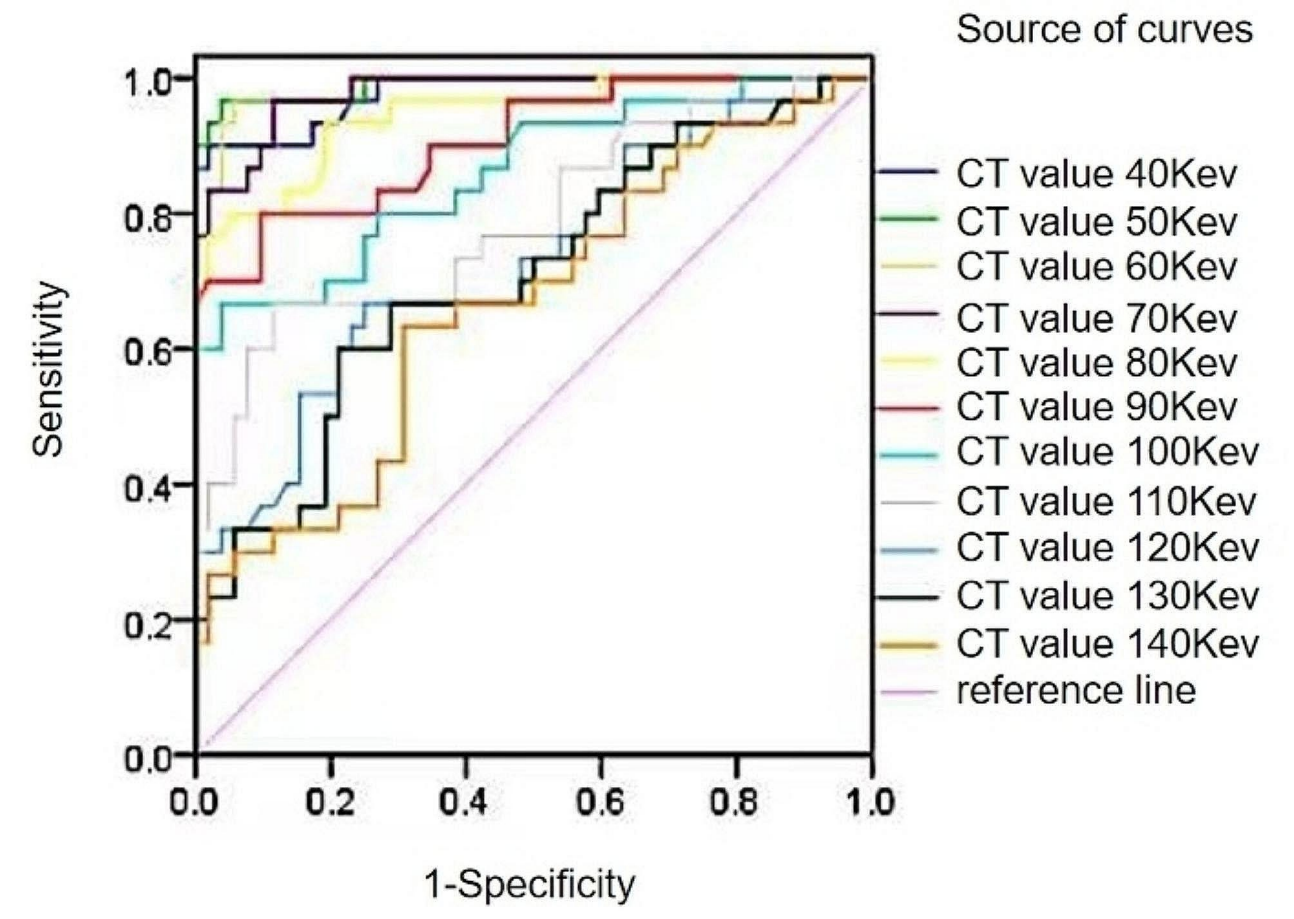
ROC curves of 40kev∼140kev single-energy CT values in the venous stage of colorectal cancer with different microvessel densities

The ROC curves of 40kev∼140kev single-energy CT values of colorectal cancer with different microvessel densities in the venous stage showed that the AUC of 40kev∼100kev single-energy CT values was greater than 0.8, which indicated that the images with 40kev∼100kev single-energy CT values in the venous stage had a better diagnostic efficacy in the difference of the microvessel densities, and the AUC of 40kev∼90kev single-energy CT values was greater than 0.9, its diagnostic efficacy was more representative. Among them, the area under the curve of 50kev single-energy CT values was the largest, which had the highest sensitivity and Youden index, and its specificity was also relatively high. See Table 9; Fig. 3.

**Fig. 4:**
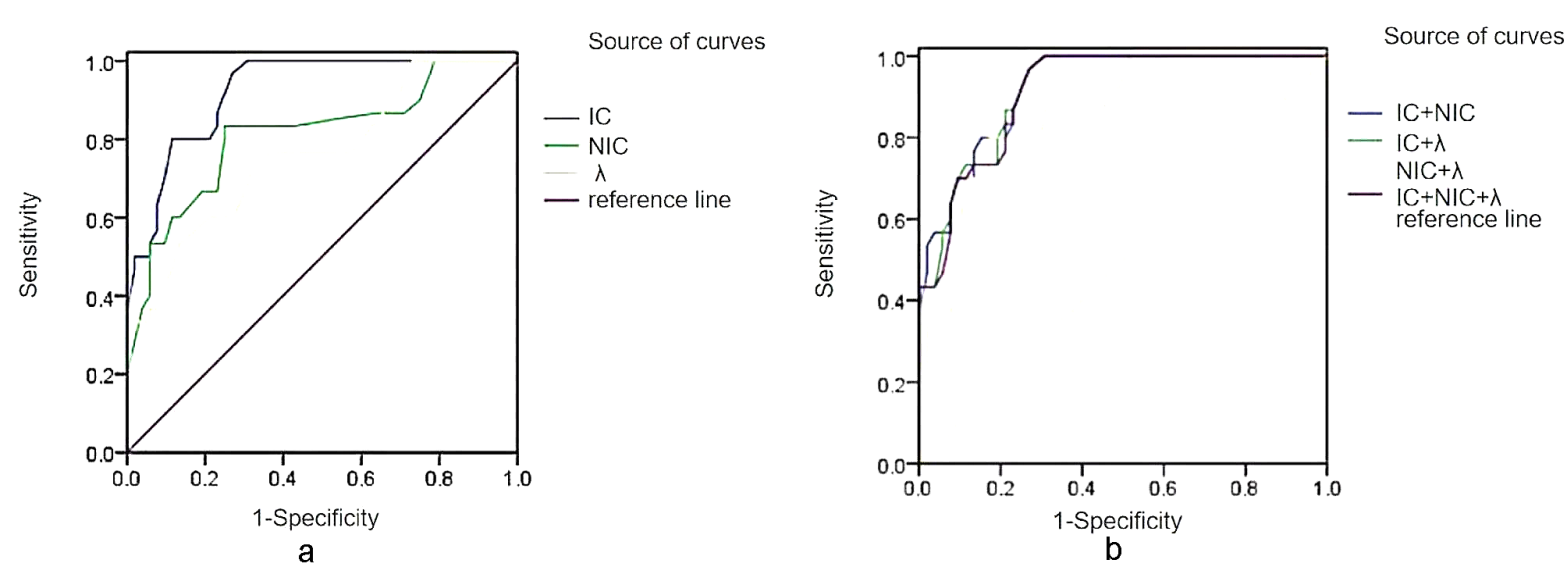
ROC curves of IC, NIC, and λ (**a**) and combined parameters (**b**) in the venous stage of colorectal cancer with different microvessel densities

The ROC curves of IC, NIC, λ and their combined parameters in the venous stage of colorectal cancer with different microvessel densities showed that the AUC of IC and NIC was greater than 0.8, indicating that the IC and NIC spectral parameters in the venous stage of colorectal cancer had very good diagnostic efficacy in the display of the differences in the microvessel densities of the colorectal cancer, the best diagnostic efficacy in the form of IC, with the highest sensitivity and specificity, as well as the Youden index. Among the IC, NIC, and λ combination parameters, the two-two combination with IC + NIC had the highest AUC of 0.922, and the sensitivity, specificity and Youden index were the same as IC. Although the combination of IC + NIC + λ has a higher diagnostic efficacy than some of the parameters alone for microvessel density in the venous phase of colorectal cancer, its diagnostic value is relatively modest compared to the overall energy spectrum parameters. See Table 10; Fig. 4.

**Table 9:** Analysis of the area under the curve of 40kev∼140kev single-energy CT values in the venous stage of colorectal cancer with different microvessel densities

Single energy level/kev	AUC(95% CI)	Threshold/HU	Sensitivity/%	Specificity/%	Youden index
40	0.977(0.917–0.998)	208.68	90.00	98.08	0.8808
50	0.990(0.937−1.000)	148.16	96.67	96.15	0.9282
60	0.987(0.932−1.000)	111.13	96.67	94.23	0.9090
70	0.978(0.918–0.998)	85.95	96.67	88.46	0.8513
80	0.944(0.870–0.983)	75.50	76.67	98.08	0.7474
90	0.907(0.823–0.960)	65.00	80.00	90.38	0.7038
100	0.859(0.765–0.926)	60.03	66.67	96.15	0.6282
110	0.789(0.685–0.871)	55.32	66.67	88.46	0.5513
120	0.728(0.619–0.821)	51.33	66.67	75.00	0.4167
130	0.705(0.595–0.801)	49.93	60.00	78.85	0.3885
140	0.663(0.550–0.764)	47.03	63.33	69.23	0.3256

**Table 10:** Analysis of energy spectral parameters and area under the curve of combined parameters in the venous phase of colorectal cancer with different microvessel densities

Spectral parameter	AUC(95% CI)	Threshold/HU	Sensitivity/%	Specificity/%	Youden index
IC	0.923(0.843–0.970)	22.30	96.67	73.08	0.6974
NIC	0.810(0.709–0.888)	0.52	83.33	75.00	0.5833
λ	0.762(0.655–0.849)	3.04	73.33	67.31	0.4064
IC + NIC	0.922(0.842–0.970)	—	96.67	73.08	0.6974
IC+λ	0.918(0.836–0.967)	—	96.67	73.08	0.6974
NIC+λ	0.862(0.768–0.928)	—	80.00	82.69	0.6269
IC + NIC+λ	0.913(0.831–0.964)	—	96.67	73.08	0.6974

*Note* IC in µgI/mL

### Analysis of CT perfusion parameters and evaluation of predictive efficacy in colorectal cancer with different microvessel densities

MVD group and 30 cases of colorectal cancer patients in the high MVD group were analyzed, statistical differences were found between the high and low MVD groups for colorectal cancer perfusion parameters BV and BF (both *P* < 0.05), and the values of BV and BF were higher in the high MVD group than in the low MVD group, while the differences in TTP, MTT and PS were not statistically different (all *P* > 0.05). See Table 11.

**Table 11:** Comparison of perfusion parameters of CT of colorectal with different microvessel densities ($\overline x \; \pm \;s$)

Perfusion parameters	Low MVD group/(*n* = 52)	High MVD group/(*n* = 30)	t/Z	*P*
BV	6.50 ± 1.58	7.81 ± 1.34	−3.830	0.001
BF	149.16 ± 10.86	197.27 ± 35.95	−8.999	0.001
TTP	29.35 ± 8.78	27.01 ± 7.34	1.229	0.223
MTT	10.48 ± 3.06	9.79 ± 2.55	1.045	0.299
PS	32.91 ± 9.81	32.04 ± 7.24	0.546	0.586

One-way logistic regression analysis of BV and BF showed that BV and BF were statistically significant in predicting microvessel density in colorectal cancer (both *P* < 0.05); The ORs of the BV and BF parameters were all greater than 1, indicating that the probability of predicting a high or low microvessel density in colorectal cancer was also relatively high for each 1-unit increase in each parameter respectively: See Table 12

**Table 12:** One-way Logistic regression analysis of CT perfusion parameters for colorectal cancer with different microvessel densities

Parameters	$\widehat{\varvec{\beta }}$	SE	Wald${\varvec{\chi }}^{2}$	*P*	OR	95% CI
BV	0.632	0.192	10.787	0.001	1.880	1.290–2.741
BF	0.374	0.116	10.499	0.001	1.453	1.158–1.823

**Fig. 5:**
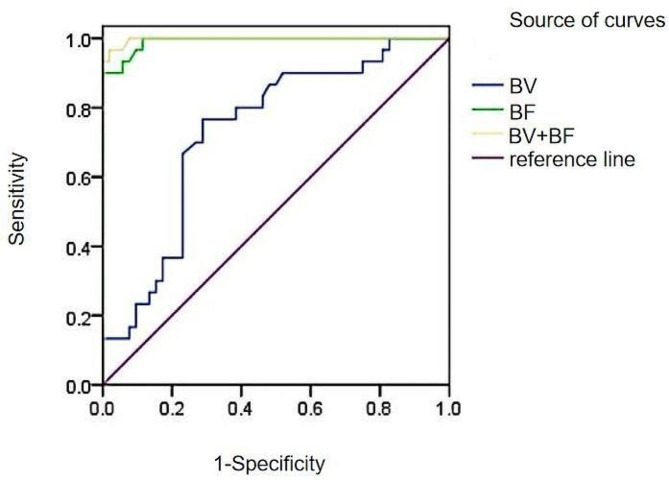
ROC curves of BV, BF and combined BV + BF parameters in colorectal cancer with different microvessel densities

ROC curves were plotted for different microvessel density colorectal cancer CT perfusion parameters BV, BF and combined BV + BF parameters, and the results showed that the AUC of BF was higher at 0.991, which had better diagnostic efficacy. the diagnostic value of colorectal cancer with different microvessel densities was high, and the sensitivity was 90.00%, the specificity was 100.00%, and the Jordon’s index was 0.9000 at the threshold value of 168.95 mL/(100 g-min), respectively. The AUC of BV was 0.733, although it has some predictive value in identifying high and low microvessel densities in colorectal cancer, its diagnostic efficacy is low compared to BF. By combining BV and BF, the AUC of the combined parameter was obtained to be 0.997, which had a high predictive efficacy in determining the high or low microvessel density in colorectal cancer. See Table 13; Fig. 5.

**Table 13:** Analysis of CT perfusion parameters and area under the curve of combined parameters for colorectal cancer with different microvessel densities

Perfusion parameters	AUC(95% CI)	Threshold/HU	Sensitivity/%	Specificity/%	Youden index
BV	0.733(0.623–0.824)	7.39	76.67	71.15	0.4782
BF	0.991(0.940−1.000)	168.95	90.00	100.00	0.9000
BV + BF	0.997(0.950−1.000)	—	96.67	98.08	0.9474

## Discussions

At the time of tumorigenesis, its diameter is generally less than 2 mm, the diffuse supply of the native tissue was sufficient to support its growth and reproduction, but in tumor growth and development, in order for the tumor to grow beyond the limited volume of 2 mm, the tumor cells must not only proliferate, but also be able to obtain a greater supply of energy from the host in order to induce new capillarisations, thus neovascularization of the tumor tissues plays a crucial role. As early as the end of the 20th century, it was proposed that tumor growth is dependent on angiogenesis, that tumor cells and blood vessels constitute a highly integrated ecosystem, that endothelial cells can be transformed from a quiescent state to a state of rapid growth by diffusion signals from tumor cells [7]. In fact, there is also considerable indirect and direct evidence that tumor growth is dependent on angiogenesis and that tumor cells can produce diffusible angiogenesis-regulating molecules [8], so exploring methods to detect tumor angiogenesis has important clinical applications for tumorigenesis, progression, prognosis and efficacy assessment.

Microvessel density (MVD) has become an important measure of angiogenic capacity within tumor tissue, MVD counts are used to determine the extent of angiogenesis in tumor tissue and to develop diagnostic and therapeutic protocols for tumors [9]. MVD counts are primarily calculated by counting the number of angiogenic vessels highlighted on various immunohistochemical stains in pathologic tissue specimens [10]. However, information about MVD can only be observed and collected in an ex vivo setting after tumor resection, thus making it impossible to assess microvessel density in tumor tissue in vivo. With the continuous development of imaging technology, CT energy spectroscopy and perfusion imaging have emerged to provide a non-invasive and convenient method to evaluate tumor angiogenesis and its biological behavior. CT energy spectroscopy and perfusion imaging can reflect tumor angiogenesis and its perfusion in vivo and dynamically, which is non-invasive and reproducible.

In clinical tumor angiogenesis studies. The degree of enhancement of tissue on CT images of enhanced CT exams is proportional to the amount of contrast uptake through the region [11], so functional vascularity in the region can be assessed using CT values or contrast-related energy spectral parameters. As the contrast agent passes through the region of interest (ROI) over time, the decay of the contrast agent provides a tissue density profile, and mathematical algorithms can be used to calculate various vascular parameters. This technique was studied in solid tumors of the kidney, pancreas, liver, head and neck, and lung, the results showed a positive correlation between vascular parameters and MVD [12–14]. However, the use of CT energy spectroscopy and perfusion imaging in colorectal cancer is relatively rare, some of the studies were also only limited to the correlation between CT-related vascular parameters and MVD [15], The diagnostic value of using CT-related vascular parameters for high and low MVD techniques was less well studied. Therefore, in this study, we systematically analyzed the diagnostic value of CT energy spectrum and perfusion parameters for colorectal cancer with different microvessel densities.

### Diagnostic value of single-energy CT values and energy spectrum parameters of CT energy spectrum imaging for colorectal cancer with different microvessel densities

Evidence that neovascularization is a key event in primary colorectal tumorigenesis. For colorectal tumors, microvessel density, a direct measure of neovascularization in solid tumors, is lower in normal intestinal mucosa than in adenomas and highest in cancers. A study by Kim JW et al [16] on the correlation of parameters related to CT enhancement values and iodine content of colorectal polyps and malignant tumors with MVD using CT functional imaging found that CT enhancement values and related parameters of iodine content correlate with MVD. Mingliang Wang et al [17] assessed anti-angiogenesis in pancreatic cancer by CT energy spectroscopy imaging and showed that CT-enhanced iodine levels and MVD counts in treated pancreatic cancer tissues were lower than in the untreated group, which indicated that CT energy spectroscopic imaging could measure the iodine content in living tumor tissues, it could sensitively detect the changes of iodine content in tumor tissues [18], and at the same time could reflect the microcirculation perfusion of tumors, which has certain diagnostic value for evaluating the angiogenesis of tumor tissues in vivo. However, the above studies did not analyze the specific diagnostic efficacy of each parameter of the energy spectrum in detail. If the parameters of the energy spectrum can be analyzed in detail and more accurate parameters for judging MVD can be derived, the workload of the imaging physicians can be greatly reduced in clinical practice, and the high efficiency of the parameters can be used to determine the degree of MVD both quickly and accurately.

The results of this study showed that the 40kev-140kev single-energy CT values were higher in the MVD high-density group than in the MVD low-density group in both the arterial and venous stages of colorectal cancer, all of them were statistically significant. The arterial and venous phase energy spectral parameters, except for the water-based values, were higher in the MVD high-density group than in the MVD low-density group in terms of IC, NIC, λ and Eff-Z, all were statistically significant. The analysis of the results of the study showed that the values of the parameters in the high-density group were higher than those in the low-density group, indicating that the colorectal cancer tissues in the high-density group had richer blood flow and higher iodine content uptake, application of CT energy spectroscopic imaging technology has certain clinical application value in determining the density of microvessels in tumor tissues of colorectal cancer in vivo.

In order to clarify more clearly the effect of each energy spectrum parameter on determining the microvessel density in colorectal cancer tumor tissues, Further analysis in this study by plotting ROC curves revealed that the AUC of 40kev∼120kev single-energy CT values in the arterial phase was greater than 0.8, indicating that 40kev∼120kev single-energy CT values on CT energy-spectrum-enhanced images have high diagnostic value for distinguishing the perfusion of colorectal cancer tumor tissues, indirectly, the difference in microvessel density in tumor tissues at 40kev∼120kev single-energy CT values was also more significant, the best diagnostic efficacy was achieved with a 40 kev single-energy CT value of 0.985 and had the highest diagnostic specificity and Youden’s index (98.08%, 0.8808). The AUC of IC, NIC, and IC + NIC in the arterial phase were all greater than 0.9, suggesting that colorectal cancer tumor tissues have a greater ability to take up iodine in CT-enhanced examination, the amount of iodine in the tumor tissue allows for an accurate assessment of the density of microvessels in the living tissue, IC had the highest AUC value of 0.952 and had high sensitivity, specificity and Yoden’s index (93.33%, 90.38%, 0.8372); The AUC of IC + NIC is higher than that of IC, indicating that the AUC of IC and NIC combined with each other is greater than 0.8 for judging the CT value of colorectal cancer perfusion, which suggests that venous colorectal cancer was more superior to judging the microvessel density of microvessels by virtue of IC alone. Intravenous phase 40kev∼100kev single-energy 40kev∼100kev single-energy CT-value images have good diagnostic efficacy for determining differences in high and low microvessel densities, and the AUC of 40kev∼90kev single-energy CT-values is greater than 0.9, whose diagnostic efficacy is more representative, the 50 kev single energy CT value has the largest area under the curve, with the highest sensitivity and Youden’s index (96.67%, 96.15%), and its specificity (0.9282) is also relatively high. The AUC of IC and NIC in the venous stage were all greater than 0.8, indicating that the IC and NIC energy spectrum parameters in venous colorectal cancer have very good diagnostic efficacy in displaying the differences in the high and low microvessel densities, the diagnostic efficacy of IC was the best at 0.923 and had the highest sensitivity, specificity and Youden’s index (96.67%, 73.08%, 0.6974). Among the IC, NIC, and λ combination parameters, the two-two combination with IC + NIC had the highest AUC of 0.922, and the sensitivity, specificity, and Youden index were the same as IC. Although the combination of IC + NIC + λ in the intravenous phase has a higher diagnostic efficacy for colorectal cancer microvessel density than some of the parameters alone, its diagnostic value was relatively low compared to the overall energy spectrum parameters. Through the systematic analysis of CT energy spectrum parameters, it was found that CT energy spectrum imaging technology could reflect the hemodynamic changes in tumor tissues by analyzing the iodine content of tumor tissues through CT enhancement scanning, and could reflect the angiogenesis of tumor tissues and its blood perfusion intuitively, which is non-invasive and reproducible, and has important value for diagnosis, prognosis and efficacy assessment of tumor patients.

### Diagnostic value of CT perfusion imaging parameters in colorectal cancer with different microvascular densities

Neoangiogenesis underlies solid tumor growth and malignant progression. For tumor tissue to proliferate and metastasize, neovascularization within it must have a certain phenotype before it can be achieved [19]. Quantitative analysis of microvessels in tumor tissues by immunohistochemical staining of vascular endothelial cell antigens accurately reflects the angiogenesis of tumor tissues [20], which is an important indicator for assessing whether tumor tissues were invasive, metastatic, recurrent for evaluating therapeutic efficacy, the results have been confirmed by a large number of studies. In this study, CD34 monoclonal antibody was used to stain the microvessels, which were stained brown in the tumor tissues and were clearly visible under the microscope, so it could provide accuracy for microvessel counting.

Tumor tissues mainly originate from glands rich in capillary network, and the contrast agent has a longer retention time when passing through the capillary network in tumor tissues, which leads to an increase in BF and BV levels; BF and BV are among the most reliable parameters for evaluating the abundance of blood vessels in tumor tissues, and their levels increase continuously with the exuberant growth of vascular structures in tumor tissues [21, 22]. In this paper, the results of a study to assess angiogenesis in colorectal cancer by means of CT perfusion parameters showed that BV and BF levels were higher in colorectal cancer patients in the MVD high-density group than in the MVD low-density group. This were statistically significant; While TTP and MTT were lower in the high MVD group than in the low MVD group, PS did not differ significantly between the high and low MVD groups, the results of TTP, MTT, and PS were not statistically significant in the two groups, the reason may be the influence of the internal blood flow characteristics of the tumor tissue and the density of microvessels inside the tumor, the more neovascularization there is in the tumor tissue, the greater the density of microvessels within it, and the higher the microvessel count per unit volume, the greater the BF of its blood flow, and the greater the BV of blood volume will be. The results of Yanyan Xu et al. showed [23] a positive correlation between BF, BV and MVD expression, echoing the results of this study. Shuford RA [24] and others have also demonstrated that BV and BF are positively correlated with MVD and can more accurately reflect microvessel density in colorectal cancer. Similarly the above studies only analyzed the correlations and did not exhaustively analyze the efficacy of each parameter of perfusion. In this study, we showed the assessment value of BV and BF on angiogenesis by ROC curve assessment, and the area under the ROC curve was 0.773 and 0.991, respectively, with BF being the highest, it indicates that CT perfusion parameters have a certain reference value for assessing microangiogenesis, and can more accurately reflect the blood flow characteristics, microvessel density, and tumor angiogenesis in living tumor tissue. To some extent, it help to determine the degree of malignancy, invasion and metastatic ability of colorectal cancer tumor tissues based on CT perfusion parameters. Meanwhile, the results of the present study showed that the predictive value of MVD could reach more than 0.9 for both energy spectrum parameters and perfusion parameters, suggests that both can monitor hemodynamics in tumor tissue in response to its biological characteristics, alternatively, in cases where the two are not available at the same time, one can be chosen from among the others.

Predictive analysis of MVD of colorectal cancer foci by one-stop energy spectroscopy and perfusion CT parameters can further assess the angiogenesis of tumors and its impact on tumor ontogeny, thus guiding clinicians to rationally apply anti-angiogenic drugs to patients with tumors, thereby effectively inhibiting rapid tumor growth and metastasis, and improving patients’ prognosis and quality of survival.

## Summary

In the present study, we analyzed 40kev∼140kev single-energy CT values water-based, iodine-based, standardized iodine-based, slope of the energy spectrum curve, effective atomic number and combined energy spectrum parameters in the arterial and venous phases of colorectal cancer with different microvessel densities, derived the iodine content in the tumor tissue can reflect the hemodynamic changes in the tumor tissue and visualize the angiogenesis and its perfusion in the tumor tissue, which was non-invasive and reproducible. it was valuable for diagnosis, prognosis and efficacy assessment of tumor patients. Helpful in aiding clinical treatment planning and assessment of patient prognosis.

By analyzing BV, BF, TTP, MTT and PS in colorectal cancers with different microvessel densities, it was concluded that the perfusion parameters BV, BF and the combined parameters of the two have a certain reference value in assessing microangiogenesi, it can accurately reflect the blood flow characteristics, microvessel density and tumor angiogenesis in living tumor tissues. To a certain extent, it helps to determine the malignant degree, invasion and metastatic ability of colorectal cancer tumor tissues based on CT perfusion parameters.

## Shortcomings and prospects

The following limitations exist in this study; only two-dimensional measurements at the largest level of the tumor were performed during lesion measurements, and the representativeness of the ROIs was somewhat restriction. In addition, due to the limitation of the thickness of the intestinal wall, the accuracy of manually sketching ROIs on the normal intestinal wall is still limited by the strict quality control in this study. In view of the above limitations, it is necessary to explore more scientific, repeatable, and accurate ROI outlining methods in the future, and to further carry out comparative studies of different imaging modalities, imaging genomics [25, 26], and deep machine learning research, etc. In future research, as imaging genomics and machine learning research deepens and sample sizes increase, we may get more accurate and unexpected gains [27, 28].

## Data Availability

The datasets used and/or analysed during the current study are available from the corresponding author on reasonable request.
